# Human Synchronization Maps—The Hybrid Consciousness of the Embodied Mind

**DOI:** 10.3390/e23121569

**Published:** 2021-11-25

**Authors:** Franco Orsucci

**Affiliations:** 1 Department of Psychology, University College London, London WC1E 6BT, UK; f.orsucci@ucl.ac.uk; 2 Norfolk and Suffolk NHS Foundation Trust, Drayton High Road, Norwich NR6 5BE, UK

**Keywords:** synchronization, semiotics, information, cognitive neuroscience, psychotherapy, conversation, mapping, chimaera states, statistical dynamics, coupling

## Abstract

We examine the theoretical implications of empirical studies developed over recent years. These experiments have explored the biosemiotic nature of communication streams from emotional neuroscience and embodied mind perspectives. Information combinatorics analysis enabled a deeper understanding of the coupling and decoupling dynamics of biosemiotics streams. We investigated intraindividual and interpersonal relations as coevolution dynamics of hybrid couplings, synchronizations, and desynchronizations. Cluster analysis and Markov chains produced evidence of chimaera states and phase transitions. A probabilistic and nondeterministic approach clarified the properties of these hybrid dynamics. Thus, multidimensional theoretical models can represent the hybrid nature of human interactions.

Science is built up with facts, as a house is with stones.

However, a collection of facts is no more a science than a heap of stones is a house.

*Henri Poincaré, Science and Hypothesis*

## 1. Introduction. Complexity, Noise, and Orders

We will try to expand some theoretical outcomes of empirical and experimental research on human interactions published by our laboratories in recent years. We built an advanced multidimensional methodology for analyzing human dynamics, mainly focusing on synchronization in an embodied mind framework [1,2]. Patterns of synchronization form the foundations of the cognition [3,4] continuum between healthy and disease states [5]. Structural coupling and synchronization arise in human dynamics in many ways, including coordination in conversations: speech, movement, emotions, and physiology [6,7,8,9,10,11,12]. It is a partially self-contained setting and practices to observe and facilitate transformation in human conditions and relations. Psychotherapy has been described as “one of the most complex bio-psycho-social systems in which patterns of language, cognition, emotion, and behavior are formed and changed through the dynamics of therapist and patient interactions” [13,14]. Beyond clinical research, studying such an exceptional human dynamics environment can lead to a general model of human dynamics, comprehending the linguistic, behavioral, and physiological realms. The integration of communication, action, bodies, and environments highlights our embodied interactions’ multimodality and parallel multiactivity [15,16].

We started by focusing our studies on language. Language study is scaled in complex structures: from informational systems to mesoscopic morphological patterns to semantic and narrative streams. In verbal interactions, voice tonality, volume, pitch, cadence, rhythm, and turn-taking are relevant. Shannon [17] built the foundations of the information theory of texts and speech. His less famous work on the prediction and entropy of printed English [18] is a resource for inspiring new research. It might be interesting to consider the distribution of information and organization in different living and nonliving systems in the same perspective. In this perspective, a graph proposed by Schreiber [19] mapped scattered areas of various forms of order, entropy and knowledge still interspersed with regions of the unknown, as in old charts. Following his mapping, we can find periodic and noisy oscillations, deterministic and stochastic areas of chaos, stochastic resonance, self-organized criticality, nonlinearity, or noise. Then, there are a few other islands where a connection between our knowledge models and real-world phenomena is yet to be well established. This kind of dynamical mapping might be synchronic and diachronic, in spatial distribution and time transitions.

The structure of different systems can be known and modified through the emergence of self-organization or by external actions, by casual or planned perturbations, including measurements. Some interactions can lead to coupling between systems, and if they repeat in time, they might produce forms of synchronization. Maturana and Varela [20] considered synchronization a form of structural coupling occurring when two systems repeatedly perturb each other. “Synchronization is a nonlinear phenomenon discovered at the beginning of the scientific revolution”, and in its classical definition, synchronization refers to adjustment or entrainment in frequencies or phase of periodic oscillators due to weak interactions that lead to structural coordination between systems" [21]. This process can lead to the emergence of adaptive behavior between interacting systems. Pecora and Carroll [22], Ott, Grebogi and Yorke [23], and Pyragas [24] found that synchronization can be used to change the dynamic behavior of complex systems.

## 2. Materials and Methods. Biosemiotics Pattern Analysis

Our initial approach was different from most of the studies mentioned above. We chose a method, Recurrence Quantification Analysis–RQA [25,26], that does not generate any specific hypothesis on the form of data and does not need to consider time series produced by a dynamic system. Our primary aim was to build a statistical tool for reliable quantitative measures of the degree of organization (as expressed by the recurrence of patterns) of a flow of signs. We demonstrated how this could be performed with a relatively simple mathematical model. The analysis of the informational structure of a text (irrespective of its meaning) could unveil the hidden matching of patterns between two speaking persons. The hidden matching relates to the flow and forms of information linking partners in conversation. Through the phonetic configuration of speech, as represented in orthography, we can extract relevant patterns in the dynamic structures of human interactions.

We used Recurrence Quantification Analysis, a methodology that can reliably measure the recurrence of patterns, determinism, and entropy. Recurrence Plots (RP) were first pioneered in physics by Eckmann, Kamphorst and Ruelle [25,26]. Later, Webber, Zbilut, Giuliani and Marwan augmented this technique by identifying nonlinear variables for the quantitative assessment of RPs, thus creating RQA. Since then, RQA has been used in different areas, from molecular dynamics [27,28] to physiology [29] and bioinformatics [30,31,32]. In performing RQA, the original time series must be placed into an embedding matrix by converting the original *n* elements column vector correspondent to the symbol series into a *p*-dimensional matrix with columns as the original *X_n_* series plus its lagged copies *X_n+_*_1_, *X_n+_*_2_, *…*, *X_n+_*_1_, while *p* is the embedding dimension. The quantification of recurrences is acquired by many different ‘counts’ of repetitions within the matrix.

While testing the robustness of this methodology on written language [33], we had to set to three (letters) for dimensional embedding, as this amplifies its sensitivity while avoiding noise from low-level statistical features (for example, asymmetrical distribution of couplets of letters). We might notice that a three-letter dimension represents a mesoscopic information level in natural language, just between single letters and whole words. We will later see the theoretical implications of this seemingly technical specification. Our time series analysis used RQA and CRQA (cross recurrence) to measure the synchronization in conversations as semiotic interactions. These informational patterns represent a preverbal and a-conscious communication channel revealed by the frequent emergence of patterns of prosodic structures (such as the musicality of phoneme sequences, stereotyped words, pauses and phrases).

Other independent centers started developing research on social and clinical interactions using a similar methodology based on recurrence analysis. For example, they studied postural or verbal time series of interpersonal coordination during conversations [34,35,36]. These studies usually took one type of time series (i.e., movement, speech, or physiology) while not considering the mutual influence between different kinds of interaction. However, as human relationship dynamics are naturally hybrid, one type of interaction can influence the coupling or uncoupling of the other streams: motor, semiotic or physiological.

## 3. Results. Hybrid Couplings and Synchronizations

**Figure 1:**
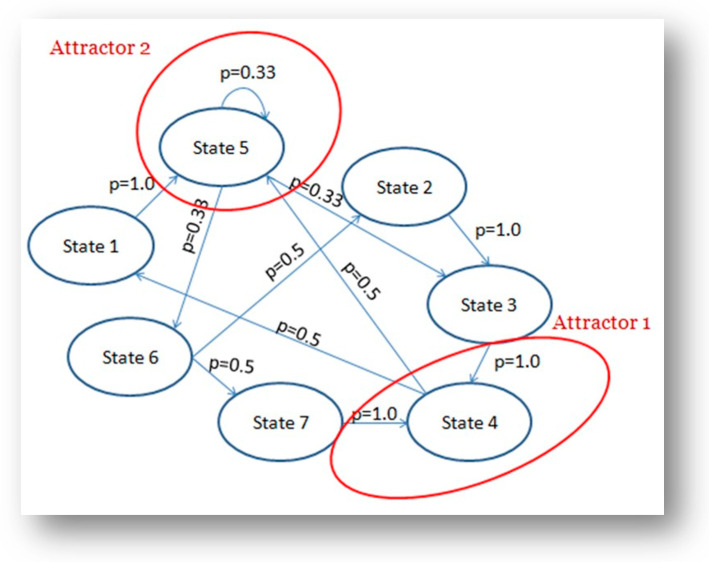
The Markov Transition Matrix map of attractors and phase transitions [1].

Human interactions constantly involve multiple streams (language, movement, emotions) which undergo coupling, decoupling and synchronizing. These multiscale and hybrid interactions are better comprehended within the biosemiotics, embodied mind framework that we defined as Mind Force [37,38]. We built the empirical paradigm of this approach as a multidimensional analysis of speech and emotions in patients and therapists in psychotherapy [39]. We chose Galvanic Skin Response—GSR and verbal prosody, as both variables reveal, in different flows, the expression of emotions [40,41]. Our new experiments studied four signals: the therapist’s speech transcription, the patient’s speech transcription, the therapist’s GSR, and the patient’s GSR. We focused on how those four variables modulated, coupled, synchronized, or desynchronized with each other. First, we considered the combinatorics and patterns of letters (phonemes) and morphemes (the minor portion of words that communicate significance). As mentioned, we had established this methodology in previous studies, which validated robust informational measures of entropy and determinism. In this new study, we initially considered the synchronization with standard correlation coefficients of Principal Components Analysis. Afterwards, we clustered all four signals using k-means resulting in a model representing this complex system’s phase space and state transitions. Then, using a Markov Transition Matrix (see Figure 1), we disclosed phase transition probabilities between linguistic and physiological time series. 

The complex dyadic system evolves between two attractors. In the first attractor, state four, the therapist strives to attune and entrain with the patient presenting low values of GSR recurrence and determinism. The therapist has high recurrence and determinism in prosody with repetitive semiotic patterns, perhaps to direct the patient’s emotional expressions. The second attractor, at state five, is characterized by a medium level of GSR recurrence and determinism for both patient and therapist. We evidence semiotic medium recurrence and determinism for the therapist and low recurrence and determinism for the patient. Overall, this phase represents a state in which the patient’s physiological anxiety becomes more manageable and linguistic expressions are more integrated. In short, while state four is an erratic phase of the interaction in which semiotics seems independent from passions, state five shows an integration. This sequence in human interactions is consistent with the literature in psychotherapy and neuroscience research on the embodied mind.

## 4. Discussion. The Chimera States in Human Interactions

This data analysis and mapping highlight dynamical landscapes of mixed states of coupling, with mixed zones of synchronization, noninteraction, and drift in uncoupling that can change over time. The Japanese physicist Yoshiki Kuramoto (1984b, 1984a) proposed a paradigmatic mathematical model to describe synchronization dynamics in a large set of coupled oscillators. The most frequent form of the model has the following equation:

(1) $$\frac{d\theta_{i}}{dt}=\omega_{i}+\frac{K}{N}\overset{N}{\underset{j=1}{\sum }}sin\left(\theta_{j}-\theta_{i}\right),\;i=1\ldots .\;N,$$

where the system is formed of *N* limit-cycle oscillators with phase *θ_i_* and coupling *K*.

Then, in November 2002, Yoshiki Kuramoto and Dorjsuren Battogtokh published the paper “Coexistence of Coherence and Incoherence in Nonlocally Coupled Phase Oscillators” [42,43]. They observed the coexistence of coherence and incoherence in a network of identical, nonlocally coupled, complex Ginzburg–Landau oscillators. While coupled nonidentical oscillators were known to exhibit mixed complex behavior (frequency locking, phase synchronization, partial synchronization, and incoherence), identical oscillators were supposed to either synchronize in phase or incoherently drift. They showed that oscillators that were identically coupled with similar natural frequencies could behave differently from one another for specific initial conditions. Some could synchronize while others remained incoherent in a stable state. They considered the following equation, which they called the nonlocally coupled complex Ginzburg–Landau equation:

$$\frac{\delta }{\delta t}\psi \left(x,t\right)=\omega \left(x\right)-\int G\left(x-x^{\prime }\right)\sin\left(\psi \left(x.t\right)-\psi \left(x^{\prime },t\right)+\alpha \right)dx^{\prime }$$

with *ω*(*x*) = *ω* for all *x*.

Later, Abrams and Strogatz [44,45] named it a chimaera state, from the mythological Greek creature made up of parts of different animals and introduced some theoretical clarifications for such behavior. Finally, they studied the most straightforward system presenting a chimaera state, a ring of phase oscillators governed by:

$$\frac{\vartheta \phi }{\theta t}\omega -\int_{-\pi }^{\pi }G\left(x-x^{\prime }\right)\sin\left[\phi \left(x,xt\right)-\phi \left(x^{\prime },t\right)+\alpha \right]dx\prime$$

Here, $\phi \left(x^{\prime },t\right)$ is the phase of the oscillator at position x at time *t.* The space variable x runs from −π to π with periodic boundary conditions. The frequency *ω* plays no role in the dynamics; one can set *ω* = 0 by redefining $\phi \underset{}{\to }\phi +\omega t$ without otherwise changing the form of the equation.

Chimaera states were later found in limit-cycle oscillators, chaotic oscillators, chaotic maps and in neuronal systems. In the beginning, chimaera patterns were observed in nonlocally coupled networks, but afterwards, these states were also found globally and locally (nearest neighbor) coupled networks and in modular networks [46,47]. The usage of Markov chains for mapping couplings and chimaera states was also explored ([48,49]. C.R. Laing studied chimaera state in heterogeneous networks, analyzing the influence of heterogeneous coupling strengths. Of further interest for human dynamics is the emergence of chimaera states in multiscale networks that result from the networking of different networks [50,51]. The ubiquity of chimaera mapping of synchronization and its different typologies extended its original definition to areas that might include nonidentical coupling oscillators in hybrid networks and multiscale networking of networks that were already known to present chimeralike dynamics before this definition started to be used.

Our studies’ dynamic mapping of heterogeneous synchronization indicates that similar dynamics involving different brain areas related to emotional, motor, and verbal interactions co-occur. Cognitive tasks constantly require a balance between segregated and integrated neural processing with relevant consequences for cognitive performance. Segregation enables efficient computations in specialized brain regions, while integrated systems ensure coordinated, robust performance. Focused states tend to involve shorter, local connections, while integration largely relies on subcortical regions and cortical hubs with diverse connections to other brain regions [52,53]. “Recognizing chimaera dynamics can help to clarify the hybrid complexity of synchronization in critical cognitive states where a balance between integration and segregation is required for adaptive cognition and social interactions” [54]. Brain chimaera dynamics might also be related to different neuronal interactions mediated by different electrical or chemical synapses in the nervous system.

Further neural interactions involve neuromodulators and hormones, faster or slower action, and different time frames [55]. Various types of neural interaction are undoubtedly an additional factor in the emergence of chimaera dynamically states in human hybrid synchronization [51]. As separate regions interact to perform neurocognitive tasks, variable patterns of partial synchrony form chimaera states [3].

## 5. Conclusions: From Determinism to Statistical Dynamics

Human dynamics are so complex and prone to indeterminacy and randomness that even deterministic chaos might be considered, in many cases, as a reductionist simplification. Therefore, we might consider probabilistic models including elements of randomness. The previous study highlighted how biopsychosocial dynamics are hybrid, discontinuous, and have many degrees of freedom. We also highlighted how cluster analysis and Markov states could help to clarify the dynamics. However, our knowledge of the state of the systems is always incomplete, and some uncertainty is part of the game. While standard dynamics usually consider the behavior of a single state, statistical dynamics define the statistical ensemble as a probability cloud of the possible conditions in the system [56]. The ensemble probability can be interpreted in two main ways:

(a) Epistemic probability of all the possible states.
(b) Empirical probability in repeated experiments.

Following this perspective, we used a probabilistic approach in the study of empathy [57]. Empathy plays a significant role in human coordination, collaboration, and change, in human interactions. Most authors agree that forms of resonance in imitation, emotions, and communication are relevant factors of empathy. Following the expanding literature on relational physiology, we explored if empathy would present physiological evidence. We applied a Principal Component Analysis (PCA) on simultaneous GSR and HR signals from a patient-therapist dyad. PCA revealed a ‘shared’ component in signals, and two ‘individual’ components of independent correlation. Regression analysis showed that the shared component predicted a therapy outcome (R^2^ = 0.28). We further examined the common component dynamics in a symbolic Markovian discrete model and cluster analysis.

Several studies on cognitive neuroscience [58,59] established statistical dynamics in biological systems focusing on the reciprocal correlations between system descriptors. This scientific position focuses on the mesoscopic level [60,61], potentially expanding correlations among system variables. This is the midpoint between pure “bottom-up” and “top-down” approaches. The crucial role of mesoscopic dynamics was validated in our physiological analysis and semiotics, as highlighted by the robust evidence for our mesoscopic embedding in RQA since the first experiments. In this way, we focused on the level of morphemes as word subcomponents. Morphemes have meaning and grammatical functions. They can be decomposed into smaller morphemes without losing these two crucial properties. Morphemes can be considered as semiotic quanta of information in natural language, as they are the basic lexical item in a language. They are usually composed of more than one phoneme and several letters or informational units [62,63,64]. Therefore, we can consider morphemes as the information quanta structuring coupling and synchronization in natural human language.

We explored informational patterns in human interactions. We investigated intraindividual and interpersonal relations as coevolution dynamics of hybrid couplings, synchronizations, and desynchronization. Cluster analyses and Markov chains produced evidence of chimaera states and phase transitions. A probabilistic and nondeterministic approach can clarify relevant properties of human dynamics, focusing on the mesoscopic scale and statistical dynamics. Theoretical models of human interactions should be founded on the hybrid nature of human structural couplings.

## References

[1] Orsucci F., Musmeci N., Aas B., Schiepek G., Reda M.A., Canestri L., de Felice G. (2016). Synchronization analysis of language and physiology in human dyads. Nonlinear Dyn. Psychol. Life Sci..

[2] Orsucci F. (2020). Towards the integration of semiotic and physiological dynamics: From nonlinear dynamics to quantum fields. Selbstorganization–Ein Paradigma für die Humanwissenschaften.

[3] Bansal K., Garcia J.O., Tompson S.H., Verstynen T., Vettel J.M., Muldoon S.F. (2019). Cognitive chimera states in human brain networks. Sci. Adv..

[4] Shine J.M., Breakspear M., Bell P.T., Martens K.A.E., Shine R., Koyejo O., Sporns O., Poldrack R.A. (2019). Human cognition involves the dynamic integration of neural activity and neuromodulatory systems. Nat. Neurosci..

[5] Hizanidis J., Kouvaris N.E., Zamora-López G., Díaz-Guilera A., & Antonopoulos C.G. (2016). Chimera-like states in modular neural networks. Sci. Rep..

[6] Glass L. (2001). Synchronization and rhythmic processes in physiology. Nature.

[7] Orsucci F., Giuliani A., Webber C., Fonagy P. (2006). Combinatorics and synchronization in natural semiotics. Phys. A Stat. Mech. Appl..

[8] Orsucci F., Petrosino R., Paoloni G., Canestri L., Conte E., Reda M.A., Fulcheri M. (2013). Prosody and synchronization in cognitive neuroscience. EPJ Nonlinear Biomed. Phys..

[9] Wiltshire T.J., Philipsen J.S., Trasmundi S.B., Jensen T.W., Steffensen S.V. (2020). Interpersonal coordination dynamics in psychotherapy: A systematic review. Cognit. Ther. Res..

[10] Tschacher W., Meier D. (2020). Physiological synchrony in psychotherapy sessions. Psychother. Res..

[11] Repp B.H., Su Y.-H. (2013). Sensorimotor synchronization: A review of recent research (2006–2012). Psychon. Bull. Rev..

[12] Delaherche E., Chetouani M., Mahdhaoui A., Saint-Georges C., Viaux S., Cohen D. (2012). Interpersonal synchrony: A survey of evaluation methods across disciplines. IEEE Trans. Affect. Comput..

[13] Schiepek G., Fricke B., Kaimer P. (1992). Synergetics of psychotherapy. Self-Organization and Clinical Psychology.

[14] Gelo O.C., Salvatore S. (2016). A dynamic systems approach to psychotherapy: A meta-theoretical framework for explaining psychotherapy change processes. J. Couns. Psychol..

[15] Mondada L. (2016). Challenges of multimodality: Language and the body in social interaction. J. Socioling..

[16] Mondada L. (2019). Contemporary issues in conversation analysis: Embodiment and materiality, multimodality and multisensoriality in social interaction. J. Pragmat..

[17] Shannon C.E., Weaver W. (1948). The Mathematical Theory of Communication.

[18] Shannon C.E. (1951). Prediction and Entropy of Printed English. Bell Syst. Tech. J..

[19] Schreiber T. (1999). Interdisciplinary application of nonlinear time series methods. Phys. Rep..

[20] Maturana H.R., Varela F.J. (1980). Autopoiesis and Cognition the Realisation of the Living.

[21] Strogatz S.H. (2003). Sync the Emerging Science of Spontaneous Order.

[22] Pecora L.M., Carroll T.L. (1990). Synchronization in chaotic systems. Phys. Rev. Lett..

[23] Ott E., Grebogi C., Yorke J.A. (1990). Controlling chaotic dynamical systems. Chaos: Soviet-American Perspective on Nonlinear Science.

[24] Pyragas K. (1996). Weak and strong synchronization of chaos. Phys. Rev. E Stat. Phys..

[25] Eckmann J.-P., Kamphorst S.O., Ruelle D. (1987). Recurrence plots of dynamical systems. Europhys. Lett..

[26] Webber C.L., Zbilut J.P. (1994). Dynamical assessment of physiological systems and states using recurrence plot strategies. J. Appl. Physiol..

[27] Giuliani A., Benigni R., Zbilut J.P., Webber C.L., Sirabella P., Colosimo A. (2002). Nonlinear signal analysis methods in the elucidation of protein sequence-structure relationships. Chem. Rev..

[28] Manetti C., Ceruso M.A., Giuliani A., Webber C.L., Zbilut J.P. (1999). Recurrence quantification analysis in molecular dynamics. Ann. N. Y. Acad. Sci..

[29] Webber C.L., Zbilut J.P. (2005). Recurrence quantification analysis of nonlinear dynamical systems. Tutor. Contemp. Nonlinear Methods Behav. Sci..

[30] Marwan N. (2008). A historical review of recurrence plots. Eur. Phys. J. Spec. Top..

[31] Webber C.L., Marwan N., Facchini A., Giuliani A. (2009). Simpler methods do it better: Success of Recurrence Quantification Analysis as a general-purpose data analysis tool. Phys. Lett. A.

[32] Webber C.L., Marwan N. (2015). Recurrence Quantification Analysis. Theory and Best Practices.

[33] Orsucci F., Walter K., Giuliani A., Webber C.L., Zbilut J.P. (1997). Orthographic Structuring of Human Speech and Texts: Linguistic Application of Recurrence Quantification Analysis. arXiv.

[34] Keller E., Tschacher W. (2007). Prosodic and gestural expression of interactional agreement. Verbal and Nonverbal Communication Behaviors.

[35] Shockley K., Richardson D.C., Dale R. (2009). Conversation and coordinative structures. Top. Cogn. Sci..

[36] Fusaroli R., Konvalinka I., Wallot S. (2014). Analyzing Social Interactions: The Promises and Challenges of Using Cross Recurrence Quantification Analysis. Translational Recurrences.

[37] Orsucci F. (2009). Mind force theory: Hyper-network dynamics in neuroscience. Chaos Complex. Lett..

[38] Freeman W.J. (2012). On the Nature and Neural Mechanisms of Mind Force. Chaos Complex. Lett..

[39] Orsucci F. (2016). Human Dynamics: A Complexity Science Open Handbook.

[40] Pichon S., Kell C.A. (2013). Affective and sensorimotor components of emotional prosody generation. J. Neurosci..

[41] Koolagudi S.G., Rao K.S. (2012). Emotion recognition from speech: A review. Int. J. Speech Technol..

[42] Kuramoto Y., Battogtokh D. (2002). Coexistence of coherence and incoherence in nonlocally coupled phase oscillators. arXiv.

[43] Smirnov L., Osipov G., Pikovsky A. (2017). Chimera patterns in the Kuramoto–Battogtokh model. J. Phys. A Math. Theor..

[44] Abrams D.M., Strogatz S.H. (2004). Chimera states for coupled oscillators. Phys. Rev. Lett..

[45] Panaggio M.J., Abrams D.M. (2015). Chimera states: Coexistence of coherence and incoherence in networks of coupled oscillators. Nonlinearity.

[46] Schöll E., Zakharova A., Andrzejak R.G. (2019). Chimera states in complex networks. Front. Appl. Math. Stat..

[47] Wang Z., Liu Z. (2020). A brief review of chimera state in empirical brain networks. Front. Physiol..

[48] Cavers M., Vasudevan K. (2015). Spatio-temporal complex Markov Chain (SCMC) model using directed graphs: Earthquake sequencing. Pure Appl. Geophys..

[49] Vasudevan K., Cavers M., Ware A. (2015). Earthquake sequencing: Chimera states with Kuramoto model dynamics on directed graphs. Nonlinear Process. Geophys..

[50] Laing C.R. (2009). Chimera states in heterogeneous networks. Chaos Interdiscip. J. Nonlinear Sci..

[51] Makarov V.V., Kundu S., Kirsanov D.V., Frolov N.S., Maksimenko V.A., Ghosh D., Dana S.K., Hramov A.E. (2019). Multiscale interaction promotes chimera states in complex networks. Commun. Nonlinear Sci. Numer. Simul..

[52] Liégeois R., Ziegler E., Phillips C., Geurts P., Gómez F., Bahri M.A., Yeo B.T., Soddu A., Vanhaudenhuyse A., Laureys S. (2016). Cerebral functional connectivity periodically (de) synchronises with anatomical constraints. Brain. Struct. Funct..

[53] Shine J.M. (2019). Neuromodulatory influences on integration and segregation in the brain. Trends Cogn. Sci..

[54] Chouzouris T., Omelchenko I., Zakharova A., Hlinka J., Jiruska P., Schöll E. (2018). Chimera states in brain networks: Empirical neural vs. modular fractal connectivity. Chaos: Interdiscip. J. Nonlinear Sci..

[55] Majhi S., Bera B.K., Ghosh D., Perc M. (2019). Chimera states in neuronal networks: A review. Phys. Life Rev..

[56] Kolmogorov A.N., Bharucha-Reid A.T. (2018). Foundations of the theory of probability, Second English Edition.

[57] Kleinbub J.R., Palmieri A., Orsucci F.F., Andreassi S., Musmeci N., Benelli E., Giuliani A., de Felice G. (2019). Measuring empathy: A statistical physics grounded approach. Phys. A Stat. Mech. Appl..

[58] Giuliani A., Tsuchiya M., Yoshikawa K. (2018). Self-organization of genome expression from embryo to terminal cell fate: Single-cell statistical mechanics of biological regulation. Entropy.

[59] Mojtahedi M., Skupin A., Zhou J., Castaño I.G., Leong-Quong R.Y., Chang H., Huang S. (2016). Cell fate decision as high-dimensional critical state transition. PLoS Biol..

[60] Freeman W.J. (2000). Mesoscopic neurodynamics: From neuron to brain. J. Physiol. Paris.

[61] Giuliani A. (2014). Networks as a privileged way to develop mesoscopic level approaches in systems biology. Systems.

[62] Feldman L.B. (1995). Morphological Aspects of Language Processing.

[63] Martinčić-Ipšić S., Margan D., Meštrović A. (2016). Multilayer network of language: A unified framework for structural analysis of linguistic subsystems. Phys. A Stat. Mech. Appl..

[64] Martin A.E., Baggio G. (2020). Modelling meaning composition from formalism to mechanism. Phil. Trans. R. Soc..

